# Rare *GBA1* genotype associated with severe bone disease in Gaucher disease type 1

**DOI:** 10.1016/j.ymgmr.2019.100544

**Published:** 2019-11-22

**Authors:** Livia d'Avila Paskulin, Rodrigo Tzovenos Starosta, Vitória Schütt Zizemer, Suélen Basgalupp, Débora Bertholdo, Filippo Pinto e Vairo, Marina Siebert, Kristiane Michelin-Tirelli, Ida Vanessa Doederlein Schwartz

**Affiliations:** a Post-Graduation Program in Genetics and Molecular Biology, Universidade Federal do Rio Grande do Sul, Porto Alegre, RS, Brazil; b Medical Genetics Service, Hospital de Clínicas de Porto Alegre, Universidade Federal do Rio Grande do Sul, Porto Alegre, RS, Brazil; c Post-Graduation Program in Medical Sciences, Universidade Federal do Rio Grande do Sul, Porto Alegre, RS, Brazil; d Hospital Moinhos de Vento, Porto Alegre, RS, Brazil; e DAPI Clinic, Curitiba, PR, Brazil; f Center of Individualized Medicine, Mayo Clinic, Rochester, MN, USA; g Department of Clinical Genomics, Mayo Clinic, Rochester, MN, USA; h Molecular and Protein Analyses Unit, Hospital de Clínicas de Porto Alegre, Universidade Federal do Rio Grande do Sul, Porto Alegre, RS, Brazil; i BRAIN Laboratory, Hospital de Clínicas de Porto Alegre, Porto Alegre, RS, Brazil

**Keywords:** Gaucher disease, *GBA1*, Bone disease, Genotype, Phenotype

## Abstract

**Introduction:**

Gaucher disease (GD) type 1 is a lysosomal disease characterised by hepatosplenomegaly, anemia, thrombocytopenia, bone changes, and bone marrow infiltration. The disease is caused by biallelic pathogenic variants in *GBA1* which codes for glucocerebrosidase, an enzyme involved in the catabolic pathway of complex lipids.

**Aims:**

To report on the case of two sisters with GD type 1 who bear a genotype never reported in the literature.

**Case report:**

Patient 1 is a 47-year-old female diagnosed at 42 years of age with chronic lumbar pain, mild splenomegaly, slightly reduced platelets and normal hemoglobin values, severe Bone Marrow Burden (BMB) score, high chitotriosidase activity, and low glucocerebrosidase. Patient 2 is a 50-year-old female, sister of patient 1, who was diagnosed after familial screening. At 45 years of age, she had osteonecrosis of the left femur and a total hysterectomy because of uncontrollable bleeding. At first evaluation, she had bone pain with a high BMB score, mild splenomegaly, normal hemoglobin, normal platelets count, elevated chitotriosidase activity, and low glucocerebrosidase activity. Both patients were found to be compound heterozygotes for the p.Glu388Lys and the p.Ser405Asn variants in *GBA1*.

**Conclusions:**

This is the first family with GD and this combination of variants which causes a phenotype remarkable for severe bone disease with no or mild hematological manifestations.

## 1 Introduction

Gaucher disease (GD) is one of the most common lysosomal disorders with an overall frequency of one in 40,000 newborns worldwide [1]; it is caused by deficient activity of *GBA1*-coded lysosomal glucocerebrosidase (acid beta-glucosidase), which leads to a building up of glucocerebroside in macrophages thereby known as “Gaucher cells”. Accumulation inside the macrophages causes malfunction and shifts their activation profile [2], ultimately leading to systemic inflammatory response [3] and symptoms such as hepatosplenomegaly, thrombocytopenia, osteonecrosis, and, in some patients, neurological deficits. GD is categorised into three types according to neurological compromising: GD type 1 is characterised by no overt neurological symptoms; GD type 2 (acute neuronopathic), by an acute and fatal neurological compromise at early age; and GD type 3 (chronic neuronopathic), by neurological compromise with onset at late childhood or at adulthood. The first specific treatment for GD was enzyme replacement therapy (ERT), with biweekly infusions of imiglucerase, velaglucerase alfa, or taliglucerase alfa. ERT offers a significant improvement for all clinical parameters, except neurological impairment. Substrate reduction therapy (SRT) with miglustat, a daily oral drug, also showed some improvement, but not as great as ERT. Eliglustat is also a SRT, and has been used as a first line therapy for GD [4,5]. GD presents a broad range of phenotypes that are partially explained by the different *GBA1* genotypes; therefore, we herein report on two sisters with a novel genotype associated with severe bone disease and mild or no hematological phenotype.

## 2 Case reports

**Fig. 1:**
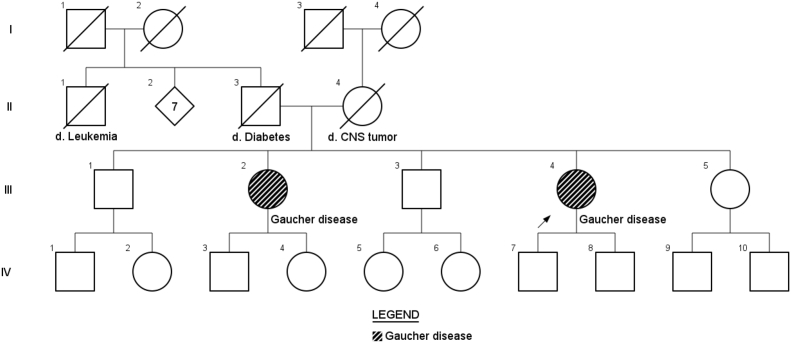
Family history. Patient 1 is the III4 and patient 2 is the III2. The individuals III1, III3 and III5 were biochemically screened negative (normal glucocerebrosidase activity).

Patient 1 is a 47-year-old female diagnosed with GD type 1 at the age of 42 years. She was born to a non-consanguineous couple and has 4 siblings of whom 3 were healthy and screened negative for GD, and one sister was symptomatic, described below as patient 2, see Fig. 1. There is no history of Parkinsonism or other neurologic symptoms in the family.

**Fig. 2:**
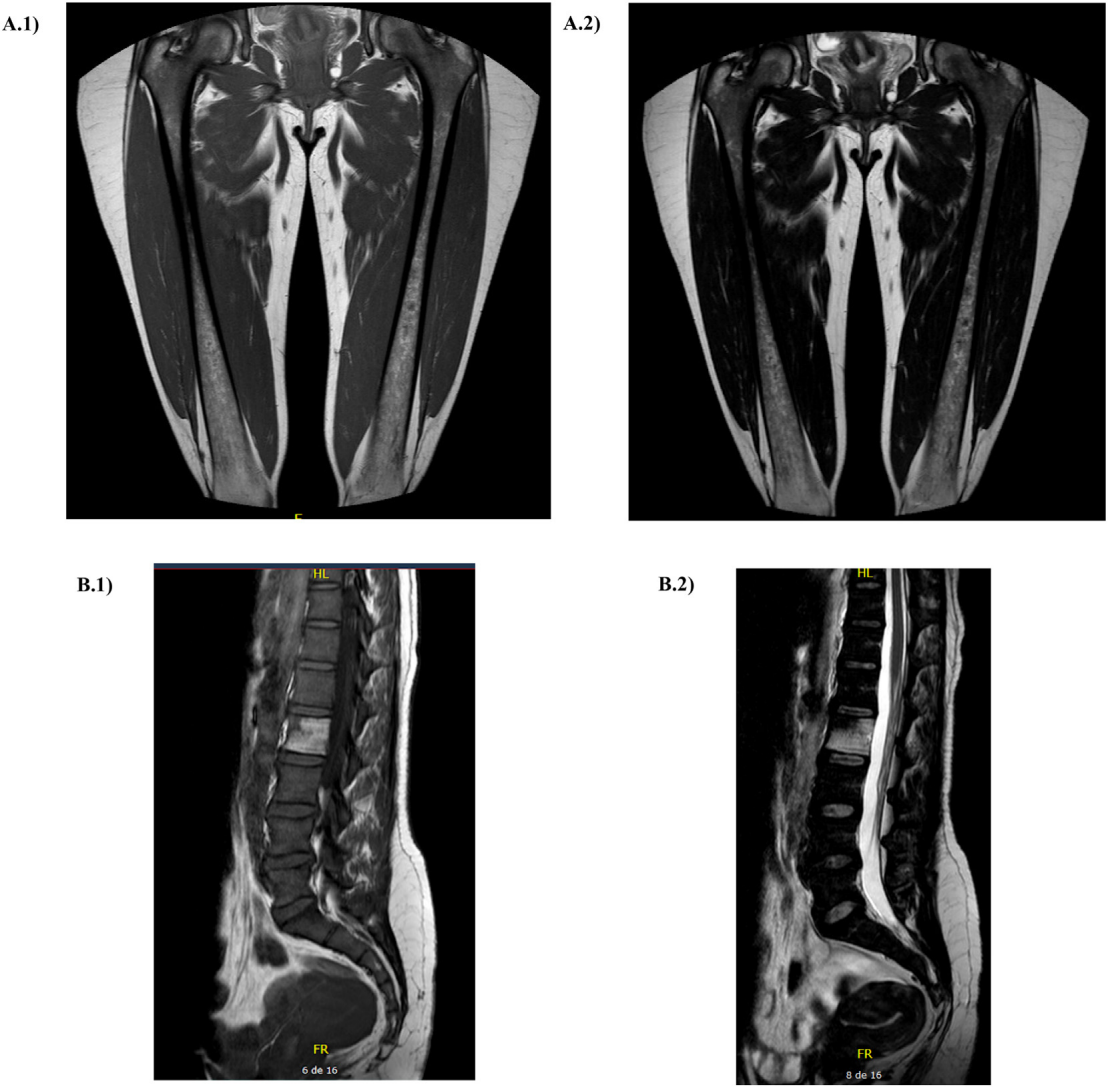
MRI images of patient 1 at baseline. A.1) Coronal T1-weighted image of femurs. A.2) Coronal T2-weighted image of femurs. B.1) Sagittal T1-weighted image of lumbar spine. B.2) Sagittal T2-weighted image of lumbar spine. Femur total score = 6 (T1 = 2, T2 = 2, Sites = 2); Lumbar spine total score = 8 (T1 = 3, T2 = 2, Pattern = 3).

She was referred to the GD Reference Center at the Hospital de Clínicas de Porto Alegre (HCPA), Brazil, because of hyperferritinaemia (ferritin = 588 ng/mL) resistant to phlebotomy treatment; chronic lumbar pain (Visual Analogue Scale = 8); and recurrent epistaxis. Laboratory exams at admission showed hemoglobin of 13 g/dL, leukocyte count at 2320 cells/mm^3^, platelets at 143,000/mm^3^, and chitotriosidase activity at 9609 nmol/h/mL (NRV = 8.8–132). Abdominal ultrasonography revealed normal liver and spleen volumes. She had normal bone metabolism markers (parathyroid hormone, alkaline phosphatase, calcium, phosphate, and vitamin D), bone mineral density (BMD) with normal Z scores, however the bone marrow burden score (BMB) was 14/16 [Fig. 2] [6]. Glucocerebrosidase activity was 5 nmol/h/mg prot in leukocytes (NRV = 10–45 nmol/h/mg prot) and 132 nmol/h/mg prot in fibroblasts (NRV = 257–688 nmol/h/mg prot) confirming the diagnosis of GD type 1. At diagnosis, the Disease Severity Scoring System [7] (DS3) was 3.6/19 (scoring only in bone subscore) and the Severity Score Index [8] (SSI) was 5/49. The patient started treatment with miglustat 300 mg/day and followed a low-carbohydrate diet. Soon after, due to diarrhea and unintended 6 kg weight loss (10% of total body weight), the patient was found to also have lactose intolerance (lactase non-persistence CC-genotype) and strongyloidiasis, and received treatment with lactose-free diet and albendazol. Due to persistence of gastrointestinal symptoms and slight clinical improvement (see Table 1), miglustat was switched after one year to taliglucerase alfa 30UI/kg/biweekly; since the patient presented an allergic reaction after 2 months of infusions to taliglucerase, it was switched to imiglucerase 30UI/kg/biweekly (see Table 1) - which regimen has been kept uneventfully, with improvement of the symptoms (Table 1).

**Table 1:** Hematological parameters, chitotriosidase activity, bone marrow burden score and DS3 subscores during treatment periods of both patients.

	Patient 1	Patient 2
***GBA1* genotype**	E349K/S366N	E349K/S366N
**Glucocerebrosidase activity**		
**Leucocytes (NRV: 10–45 nmol/h/mg protein)**	5	2.8
**Fibroblast (NRV 257–668 nmol/h/mg protein)**	132	60
**Baseline**		
Age at diagnosis (years)	42	45
Bone Marrow Burden score	13	14
Hb (g/dL)	14.6	11.6
Platelets (/μL)	143,000	192,000
Ferritin (ng/mL)	588	880
Chitotriosidase activity (nmol/h/mL)	15,581	3432
DS3 subscores		
Bone	3.6	2
Visceral	0	0
Hematological	0	0
**After 1 year of treatment**		
**Drug**	Miglustat 300 mg/day	Miglustat 300 mg/day
**Time in months of the current treatment**	12	12
Bone Marrow Burden score	13	14
Hemoglobin (g/dL)	14	11.7
Platelets (/μL)	172,000	207,000
Ferritin (ng/mL)	536	1160
Chitotriosidase activity (nmol/h/mL)	8591	1667
DS3 subscores		
Bone	3.6	2
Visceral	0	0
Hematological	0	0
**After 2 years of treatment**		
**Drug**	Imiglucerase 30 Ui/kg/inf	Miglustat 300 mg/day
**Time in months of the current treatment**	2	24
Bone Marrow Burden score	NA	14
Hemoglobin (g/dL)	14.5	11.8
Platelets (/μL)	224,000	184,000
Ferritin (ng/mL)	937	1117
Chitotriosidase activity (nmol/h/mL)	5821	1984
DS3 subscores		
Bone	3.6	2
Visceral	0	0
Hematological	0	0
**After 3 years of treatment**		
**Drug**	Imiglucerase 30 Ui/kg/inf	Taliglucerase 15Ui/kg/inf
**Time in months of the current treatment**	14	1
Bone Marrow Burden score	3	14
Hemoglobin (g/dL)	14.5	12.4
Platelets (/μL)	259,000	202,000
Ferritin (ng/mL)	608	1025
Chitotriosidase activity (nmol/h/mL)	1472	1689
DS3 subscores		
Bone	1.6	2
Visceral	0	0
Hematological	0	0
**After 4 years of treatment**		
**Drug**	Imiglucerase 30 Ui/kg/inf	Taliglucerase 15Ui/kg/inf
**Time in months of the current treatment**	36	13
Bone Marrow Burden score	NA	NA
Hemoglobin (g/dL)	15.3	13.2
Platelets (/μL)	287,000	234,000
Ferritin (ng/mL)	689	1053
Chitotriosidase activity (nmol/h/mL)	881	1133
DS3 subscores		
Bone	1.25	0
Visceral	0	0
Hematological	0	0

NA = Not Available.

**Fig. 3:**
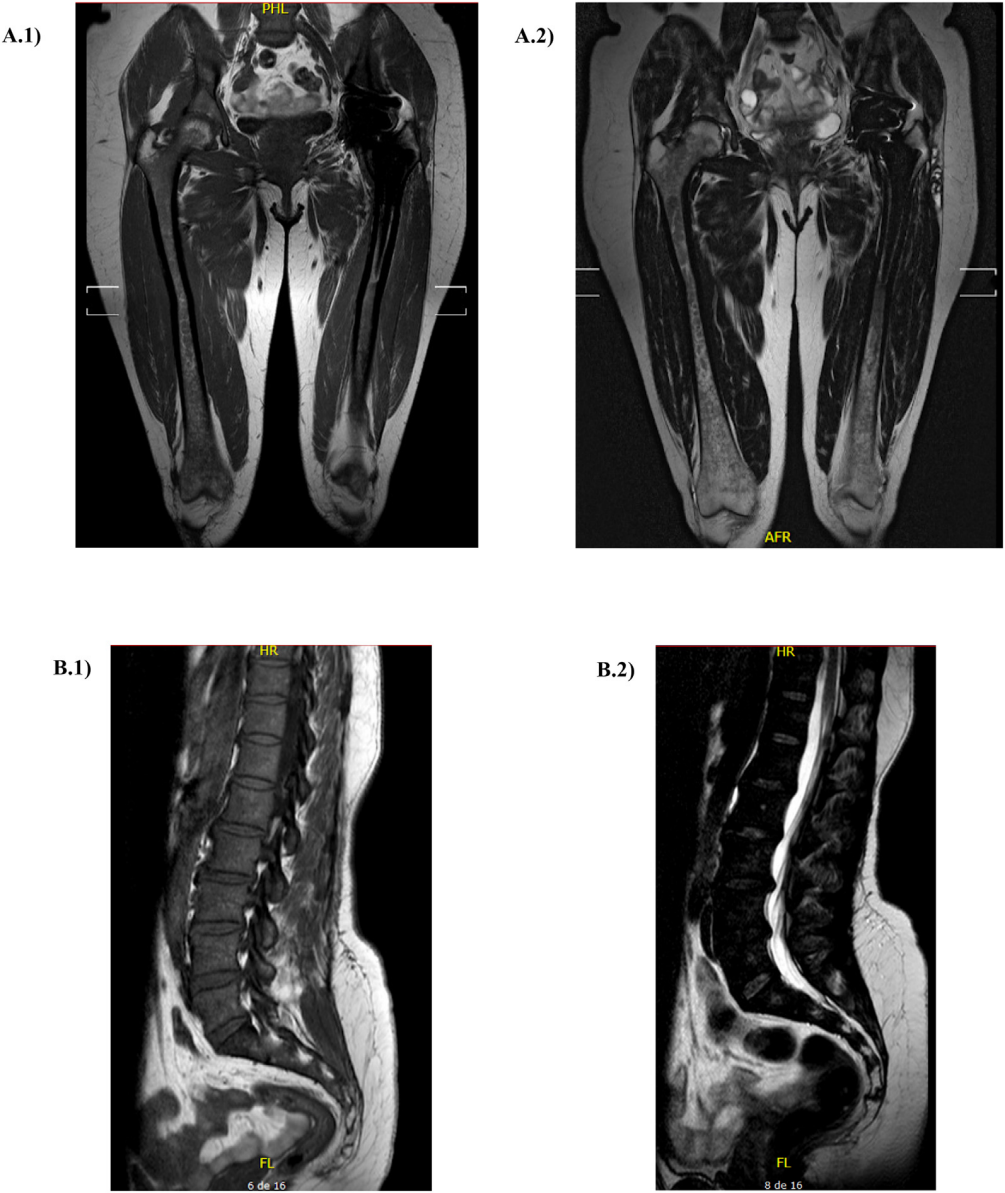
MRI images of patient 2 at baseline. A.1) Coronal T1-weighted image of femurs. A.2) Coronal T2-weighted image of femurs. B.1) Sagittal T1-weighted image of lumbar spine. B.2) Sagittal T2-weighted image of lumbar spine. Femur total score = 7 (T1 = 2, T2 = 2, Sites = 3); Lumbar spine total score = 7 (T1 = 2, T2 = 2, Pattern = 3).

Patient 2 is a 50-year-old female diagnosed with GD type 1 when she was 45 years old. Four years before the first appointment with Medical Genetics, the patient underwent a prosthetic replacement of the left femoral-acetabular joint for osteonecrosis, and, one year after, underwent total hysterectomy for uncontrollable bleeding during uterine polyp removal surgery. Laboratory tests at admission to our Center showed hemoglobin at 11.5 g/dL, leukocyte count at 8710 cells/mm^3^, platelets at 195,000/mm^3^, ferritin of 880 ng/mL, and chitotriosidase activity of 2970 nmol/h/mL (NRV = 8.8–132). Further investigation revealed mild hepatosplenomegaly and hepatic steatosis by abdominal ultrasonography. She had normal bone metabolism markers, BMD with normal T scores and BMB of 14/16 [Fig. 3]. Glucocerebrosidase activity of 2.8 nmol/h/mg protein in leukocytes (NRV = 10-45 nmol/h/mg protein) and 60 nmol/h/mg protein in fibroblasts (NRV = 257-688 nmol/h/mg protein) confirmed the diagnosis of GD. The severity scores were DS3 = 2/19 (scoring only in bone subscore) and SSI = 1/49. Because of needle phobia, she started on treatment with miglustat 300 mg/day together with a low-carbohydrate diet.

After 3 years of treatment with miglustat with unsatisfactory response (Table 1), treatment was switched to taliglucerase alfa 15UI/kg/biweekly. After 2 years of treatment with ERT, the hematological parameters and chitotriosidase activity improved, however ferritin remained high and bone marrow infiltration remained severe.

Upon genotyping with next-generation sequencing (NGS) both patients 1 and 2 were discovered to be compound heterozygotes for c.1162G>A (p.Glu388Lys) (E349K) and c.1214G>A (p.Ser405Asn) (S366N) *GBA1* pathogenic variants. Both patients tested negative for the chitotriosidase gene (CHIT1) null variant. Patients are also heterozygote for the *HFE1* pathogenic variant c.187C>G (p.His63Asp).

## 3 Discussion

### 3.1 Genotype

The patients described herein were compound heterozygotes for two uncommon *GBA1* pathogenic variants, E349K and S366N. The former has been previously described by Grabowski and colleagues in 2006 [9]; however, no clinical phenotype description nor if it was in homozygosis or compound heterozygosis with a different variant was provided. The latter, was described in compound heterozygosis with R48W (p.Arg87Trp) by Demina and Beutler in 1998 [10] in an African-American female GD type I1patient whose sister had anemia, mild thrombocytopenia, mild neutropenia, and moderate hepatosplenomegaly – however, no more details on the patient's phenotype are provided. The E349K residue is on a coil motif at the eighth exon, in a region of neutral hydropathy. This variant is predicted to cause a reduction of 88% of the normal enzyme activity [11]. The S366N variant lays on an alpha-helix at the 3′ end of the eighth exon, in a region of neutral hydropathy, and impairs a phosphorylation site. Of note, the combination of these variants in our patients caused enzyme activity higher than expected for classical GD patients.

Both variants are considered pathogenic when applying the ACMG [12] classification criteria: they are absent from gnomAD (PM2), were previously detected in trans with a pathogenic variant (PM3), multiple in silico algorithms (such as DANN, FATHMM-MKL, SIFT, LRT, MutationTaster) predict both variants to be deleterious (PP3), patient's phenotype and family history are highly specific for GD (PP4), UniProt classifies this variant as ‘disease’ (PP5), and the variant segregates with the phenotype in a gene definitively known to cause the disease (PP1).

## 4 Phenotype

Much is being studied about secondary modifier genes in Mendelian disorders, including GD [[13], [14], [15]]; however, still little is known about how strong is the genotype-phenotype association in GD. In the presented case, both patients harboured the same variants in *GBA1*, and although quite similar overall, there were some differences between the two sisters' phenotypes: while patient 2's bone phenotype may be considered somewhat more severe, patient 1's chitotriosidase – a biomarker for GD activity – was more than three times higher at admission than patient 2's. Liver and metabolic profiles, on the other side, were quite similar. This perhaps may be explained by the action of an unidentified modifier gene harboured by only one of the patients, or it may be due to environmental factors.

Osteonecrosis is a common manifestation of GD, with up to one third of GD patients experiencing it [16,17]. The most common site affected is the femoral head [18], as was the case of patient 2. In a study published by the International Collaborative Gaucher Group (ICGG) searching for risk factors for osteonecrosis [16], the only identified ones were anemia and splenectomy. Being their genotype for *GBA1* the same and neither having been submitted to splenectomy, we cannot but wonder whether patient 2 being anemic at admission was related to her having had osteonecrosis, and her sister, which was not anemic at admission, having it not.

Another common hallmark of GD is bone marrow infiltration, which can be best assessed through the method of Dixon quantitative chemical shift (Dixon's QCSI) [19], but unfortunately this method is not available worldwide. Because of that, different other semiquantitative methods are worldwide used to measure the bone marrow infiltration [6,20]. The method that correlates the best with the Dixon's QCSI method, and evaluates both the axial and the peripheral skeleton is the MRI-based BMB score published by Mass et al., which relies on signal intensity as a measure of fat substitution for Gaucher cells in the bone marrow of femurs and lumbar spine [6]. Bone manifestations of GD are secondary to Gaucher cells infiltration in the bone marrow, together with possible phenotype modifiers genes [21]. What constitutes severe bone disease in GD is open to debate. Although both sisters presented with a normal BMD and no fractures, and only one had hip necrosis, both had a severe BMB score, which may imply a more severe bone phenotype caused by the unusual combination of the E349K/S366N *GBA1* variants. Besides that, as the DS3 subscores show, for both patients the compromise of bone is more severe than the compromise of visceral and hematological systems. Also, the pattern of decrease of the BMB score during treatment shows that patient 1, who was being treated with ERT for 24 months, presented a fast response with a significantly drop in the total score when compared to patient 2, who was beign treated with SRT for 24 months and only 1 month with ERT. This is in accordance with previous studies that have shown that BMB tend to decrease during the first years of ERT, but this cannot be observed with SRT, and, also, the response is not known to reflect disease severity [22,23].

At admission, neither patient was profoundly thrombocytopenic nor anemic. Nor did the patients present overt hepatosplenomegaly, although patient 2 had mild hepatosplenomegaly and mild hepatic steatosis. Overall, the patients could be described as having predominantly severe bone disease and few, mild visceral and hematological manifestations. Whether this is due to environmental factors or indeed to the patients' rare genotype is still unclear, and more reports of patients with the same *GBA1* genotype are needed before a conclusion may be confidently drawn. Response to substrate reduction therapy with miglustat was not satisfactory for both sisters, whereas response to enzyme replacement therapy was satisfactory regarding hematological and visceral parameters; both patients reached their goals following the Brazilian Guideline [24] and the European Working Group on Gaucher Disease in 2018 [25].

Mehta *et al* published in 2019 [26] the presenting signs and patient co-variables in Gaucher disease, and highlighted that physicians can fail to recognise the early stages of GD, which can lead to significant diagnostic delays and sometimes irreversible but avoidable morbidities. When it comes to a classic GD phenotype with massive splenomegaly, bone pain and cytopenias, diagnosis is more intuitive. On the other hand, if the patient has mild symptoms, or, as in our patients' cases only bone disease, the diagnosis becomes trickier and less intuitive, requiring greater expertise to be defined.

## 5 Conclusions

This is the first GD family with the E349K/S366N *GBA1* genotype which is associated with severe bone disease and mild visceral and hematological manifestations. More genotype-phenotype studies are needed to fully establish a causational relationship between this rare genotype and the patients' unique phenotype.

## Declaration of competing interest

The authors declare no conflict of interest.
